# Corynoxine Protects Dopaminergic Neurons Through Inducing Autophagy and Diminishing Neuroinflammation in Rotenone-Induced Animal Models of Parkinson’s Disease

**DOI:** 10.3389/fphar.2021.642900

**Published:** 2021-04-13

**Authors:** Leilei Chen, Yujv Huang, Xing Yu, Jiahong Lu, Wenting Jia, Juxian Song, Liangfeng Liu, Youcui Wang, Yingyu Huang, Junxia Xie, Min Li

**Affiliations:** ^1^ Institute of Brain Science and Disease, Qingdao University, Qingdao, China; ^2^ Shandong Provincial Collaborative Innovation Center for Neurodegenerative Disorders, Qingdao University, Qingdao, China; ^3^ Shandong Provincial Key Laboratory of Pathogenesis and Prevention of Neurological Disorders, Qingdao University, Qingdao, China; ^4^ Mr. and Mrs. Ko Chi Ming Centre for Parkinson’s Disease Research, School of Chinese Medicine, Hong Kong Baptist University, Kowloon Tong, Hong Kong; ^5^ State Key Laboratory of Quality Research in Chinese Medicine, Institute of Chinese Medical Sciences, University of Macau, Macau, China; ^6^ Medical College of Acupuncture-Moxibustion and Rehabilitation, Guangzhou University of Chinese Medicine, Macau, China

**Keywords:** parkinson’s disease, corynoxine, rotenone, autophagy, α-synuclein, neuroinflammation

## Abstract

Recent studies have shown that impairment of autophagy is related to the pathogenesis of Parkinson’s disease (PD), and small molecular autophagy enhancers are suggested to be potential drug candidates against PD. Previous studies identified corynoxine (Cory), an oxindole alkaloid isolated from the Chinese herbal medicine *Uncaria rhynchophylla* (Miq.) Jacks, as a new autophagy enhancer that promoted the degradation of α-synuclein in a PD cell model. In this study, two different rotenone-induced animal models of PD, one involving the systemic administration of rotenone at a low dosage in mice and the other involving the infusion of rotenone stereotaxically into the *substantia nigra* pars compacta (SNpc) of rats, were employed to evaluate the neuroprotective effects of Cory. Cory was shown to exhibit neuroprotective effects in the two rotenone-induced models of PD by improving motor dysfunction, preventing tyrosine hydroxylase (TH)-positive neuronal loss, decreasing α-synuclein aggregates through the mechanistic target of the rapamycin (mTOR) pathway, and diminishing neuroinflammation. These results provide preclinical experimental evidence supporting the development of Cory into a potential delivery system for the treatment of PD.

## Introduction

Parkinson’s disease (PD) is the second most common neurodegenerative disease, affecting more than 1% of the population over the age of 60 years. In 2005, it was predicted that the number of individuals (age > 50 years) with PD would double by 2030, and this number increased by approximately 10% in 2018 (Rossi et al., 2018). Usually, both selective degeneration of dopaminergic neurons in the *substantia nigra* pars compacta (SNpc) and the appearance of Lewy bodies, whose main component is aggregated α-synuclein, in the remaining neurons are thought to be the major pathological hallmarks of PD (Przedborski, 2017; Johnson et al., 2019). Although the pathogenic factors of PD have been comprehensively investigated since the first detailed description by James Parkinson in 1817, the pathogenesis of this disease has not been fully elucidated and there are no effective drugs for its cure.

The autophagy–lysosome pathway is one of the major pathways that clear disordered, especially long-lived proteins, such as α-synuclein (Hou et al., 2020). Impaired autophagy is thought to exacerbate the aggregation of α-synuclein, thereby contributing to the pathological development of PD in patients and animal models (Xilouri et al., 2016; Chen et al., 2019; Hou et al., 2020). Therefore, autophagy enhancers, which could promote the clearance of aggregated α-synuclein, are suggested to be a new therapeutic measure for PD. A previous study conducted by our research group identified corynoxine (Cory), an indole alkaloid isolated from the Chinese herb *Uncaria rhynchophylla* (Chen et al., 2014; Chen et al., 2017), as a new autophagy enhancer that promoted the clearance of α-synuclein in a cell model of PD. However, the microenvironment between *in vitro* and *in vivo* cells is quite different, and the information obtained from cells models is limited. Therefore, it is necessary to evaluate the neuroprotective properties of Cory in animal models of PD prior to commencing clinical trials.

The impairment of mitochondrial complex I was reported to be a major factor that contributed to neurodegeneration (Betarbet et al., 2000). Consequently, inhibitors of mitochondrial complex I, such as rotenone (Xiong et al., 2012; Miyazaki et al., 2020), are widely used to reproduce Parkinson-like symptoms in animals. In this study, two animal models of PD were employed, one of which involved stereotaxically injecting rotenone into the SNpc of rats to establish rat models of PD with acute toxicity (Xiong et al., 2009; Anusha et al., 2017), and the other one involved systemically exposing C57BL/6J mice to a low dose of rotenone to establish mouse models of PD with chronic toxicity (Miyazaki et al., 2020). The neuroprotective effects of Cory in the two PD models were evaluated, and the results provided experimental evidence to support the development of Cory for use in the treatment of PD.

## Materials and Methods

### Reagents

Apomorphine (Y0001465) and rotenone (R8875) were purchased from Sigma; Cory was purchased from Aktin Chemicals Inc. (Chengdu, China); anti-TH antibody (AB152) was purchased from Merck Millipore; anti–Iba-1 (019-19741) was purchased from Wako Pure Chemical Industries; anti–β-actin (sc-47778) was purchased from Santa Cruz Biotechnology; anti-LC3 (2775), anti-p62 (5114), anti–phospho-p70S6K (Thr389) (9234), anti–phospho-mTOR (Ser2448) (2971), and anti–α-syn (2628) were purchased from Cell Signaling Technology; goat anti-rabbit IgG (H + L) secondary antibody, Alexa Fluor® 594 conjugate (R37117), goat anti-mouse (626520), and goat anti-rabbit (G21234) secondary antibodies were purchased from Invitrogen; and VECTASTAIN Elite ABC Kit (PK-6101) was purchased from Vector Lab. ELISA kits were purchased from Beijing 4A Biotech Co., Ltd.

### Animals

C57BL/6J mice (25∼30 g) and SD rat (220∼250 g) were purchased from Beijing Weitong Lihua Experimental Animal Technology Co., Ltd.

### Dopamine Level Detection

Detection of the dopamine level in the striatum is followed with previous report (Li et al., 2018). Briefly, striatum was weighted and homogenized in 0.5 ml ice cold 0.1 M HCl, 30 s, 50 Hz. After adding 1 ml methanol with 0.1 M HCl, homogenization was performed at 50 Hz, 1 min. Lysates were centrifuged 16,000 rpm, for 10 min at 4°C. 1 ml supernatant was taken out to freeze-dry for 48 h. Then, 30 µl 0.1 M HCl was added to dissolve the sample. 70 µl methanol with 0.1 M HCl was added to precipitate the protein. Centrifugation with 16,000 rpm for 10 min at 4°C was performed. The supernatant was subject to be analyzed with liquid chromatography (Agilent 1290, San Jose, CA, United States) coupled with electrospray ionization on a triple quadrupole mass spectrometer (Agilent 6460, San Jose, CA, United states).

### TH Immunostaining

The protocol is followed the VECTASTAIN Elite ABC Kit. Working solution was prepared before staining. Frozen brain sections (SNpc: 30 μm, striatum: 25 μm) were prepared with Cryostat Series (7721.160 GB, SHANDON). Sections were rinsed with PBS for 3 times (3 min/time). After incubating with 3% H_2_O_2_ for 10 min, sections were rinsed with PBS for another 3 times (3 min/time). Then, sections were blocked with normal serum for 30 min, and rinsed with PBS for 3 times (3 min/time). Sections were incubated with primary antibody (1:500) overnight at 4°C. Then, after rinsing with PBS for 3 times (3 min/time), sections were incubated with Elite second antibody for 30 min at room temperature. After rinsing with PBS for 3 times (3 min/time), sections were incubated with Elite ABC reagent for another 30 min at room temperature. Sections were rinsed with PBS for 3 times (3 min/time), and incubated with DAB reagent for 2 min. At last, the slides were mounted and images were taken with confocal.

### Fluoresce Immunostaining

Working solution was prepared before staining. Frozen brain sections (SNpc: 30 μm, striatum: 25 μm) were prepared with Cryostat Series (7721.160 GB, SHANDON). Sections were rinsed with PBS for 3 times (3 min/time). After incubating with 3% H_2_O_2_ for 10 min, sections were rinsed with PBS for 3 times (3 min/time). Sections were blocked with normal serum for 30 min and rinsed with PBS for 3 times (3 min/time). Sections were incubated with primary antibody overnight at 4°C. Then, sections were rinsed with PBS for 3 times (3 min/time) and incubated them with Alexa Fluor 594 s antibody for 30 min at room temperature. At last, sections were rinsed with PBS for 3 times (3 min/time), slides were mounted, and images were taken with confocal.

### Western Blotting Analysis

Tissues were lysed with RIPA lysis buffer (150 mM NaCl, 50 mM Tris-HCl, 0.35% sodium deoxycholate, 1 mM EDTA, 1% NP40, 1 mM PMSF, 5 mg/ml aprotinin, and 5 mg/ml leupeptin). The boiled samples (each containing 10–20 μg of protein) were subjected to SDS-PAGE on a 10–15% acrylamide gel and transferred to PVDF membranes (GE Healthcare, RPN303F). The membranes were blocked for 1 h in TBST containing 5% nonfat milk and then probed with the appropriate primary and secondary antibodies. The desired bands were visualized using the ECL kit (Pierce, 32106). The band density was quantified using the ImageJ program and normalized to that of the control group.

### Statistical Analysis

Statistical significance was assessed by using one-way ANOVA with the Newman–Keuls’ multiple comparison tests. Calculations were performed using ImageJ and Prism (version 5) software. Statistical significance was considered when *p* < 0.05.

## Results

### Determining the Toxicity of Cory

At the beginning of this study, an acute toxicity study was performed to confirm the safe dosage of Cory. Five female Sprague–Dawley (SD) rats (8 weeks old) weighing 200 ± 10 g were used in the study. The rats were placed separately in laboratory animal houses at 20∼25°C with 50∼60% humidity and 12 h light/dark cycle with the lights off at 7 PM. The rats were fed with standard diet from Lab Diet, allowed to access distilled water ad libitum, and acclimated to laboratory conditions for 7 days. Test up-and-down procedure followed the OECD guideline. Acute Oral Toxicity (Guideline 425) Statistical Program (AOT425StatPgm) developed by the US Environmental Protection Agency was used. The assumed dose progression factor of 1.2 was used in the study. Whether the dosing will continue depends on the 48-h outcomes after dosing. Test will be stopped when one of the following stopping criteria first is met: 1) 3 consecutive animals survive at the upper bound; 2) 5 reversals occur in any 6 consecutive animals tested; or 3) at least 4 animals have followed the first reversal and the specified likelihood ratios exceed the critical value.

Cory was directly dissolved in the normal saline. The final injection volume for each rat was 1.0 ml. Before dosing, each rat was fasted overnight. It has been reported that the intravenous (IV) dose required to kill half the members of a tested population after a specified test duration (LD_50_) of rhynchophylline, which is an isomer of Cory, is 105 mg/kg in mice (Ozaki, 1989). Therefore, based on this information, the limit test using a dosage of 2000 mg/kg was skipped, and the main test with first dosage of 80 mg/kg was performed immediately. The data generated from this study (Table 1) show that the IV LD_50_ of Cory in rats was approximately 96.89 mg/kg, which was calculated by the Acute Oral Toxicity (Guideline 425) Statistical Program (AOT425StatPgm).

**TABLE 1:** Experiment data of Cory acute toxicity.

Test sequence	Animal ID	Dose (mg/kg)	Short-term result	Long-term result
1	1	80	O	O
2	2	105	X	X
3	3	80	O	O
4	4	105	X	X
5	5	80	O	O

O, survived; X, died; short-term, 48 h; long-term, 14 days.

### Establishing the Rotenone-Induced Acute and Chronic Toxicity Models of PD

In this study, both the rotenone-induced acute toxicity rat model and rotenone-induced chronic toxicity mouse models of PD were employed to evaluate the neuroprotective effects of Cory. The classic mTOR inhibitor, rapamycin, acted as a positive control of autophagy induction, and Sinemet® and Madopar®, which are widely used in clinical practice (Chen and Xie, 2018a), acted as positive controls for the treatment of PD. Every tablet of Sinemet® contains 200 mg of levodopa and 50 mg of carbidopa, and every tablet of Madopar® contains 200 mg of levodopa and 50 mg of benserazide.

The rotenone-induced acute toxicity rat models of PD were established in male SD rats that were 8 weeks old. The SD rats were randomly divided into 5 groups (16 rats/group). The detailed groups were as follows: vehicle control group (Vehicle group), rotenone-induced PD rat group (Rotenone group, 3 μg/μl * 1 μl), Sinemet® group (Rot + Sinemet group, 0.975 mg Sinemet/rat), low-dose Cory group (Rot + Cory-L, 2.5 mg/kg), and high-dose Cory group (Rot + Cory-H group, 5 mg/kg). Rotenone (3 μg/μl) was dissolved in dimethyl sulfoxide (DMSO) and was kept away from light before use. According to Bao’s rat cerebral stereotaxic atlas, rotenone (1 μl) was injected into the right-side substantia nigra compacta (SNpc) (AP: −5.3 mm; ML: 2.0 mm; DV: −8.0 mm). The Sham/Vehicle group was injected with the same amount of DMSO. Cory or Sinemet® was orally administered to the rats based on their group allocations. Before the injection of rotenone, rats were pretreated with Cory or Sinemet® for 1 week, and the treatment was continued for 4 weeks after the model was established.

The chronic toxicity models of PD were established in 10-week-old male C57BL/6J mice that were orally administered with rotenone for 8 weeks. The C57BL/6J mice were randomly divided into 6 groups (20 mice/group). The detailed groups were as follows: the vehicle control group (Vehicle group), rotenone-induced PD mice group (Rotenone group, 30 mg/kg), Madopar® group (Rot + Madopar group, 1.95 mg Madopar/mouse), rapamycin group (Rot + Rapamycin group, 10 mg/kg), low-dose Cory group (Rot + Cory-L, 5 mg/kg), and high-dose Cory group (Rot + Cory-H group, 10 mg/kg). All of the drugs or compounds, including Cory, were orally administered to the C57BL/6J mice. Before the administration of rotenone, the mice were pretreated with Madopar®, rapamycin, or Cory for 1 week, and the administration coupled with rotenone was continued for another 8 weeks.

### Effects of Cory on Motor Dysfunction in the Rotenone-Induced Animal Models of PD

**FIGURE 1:**
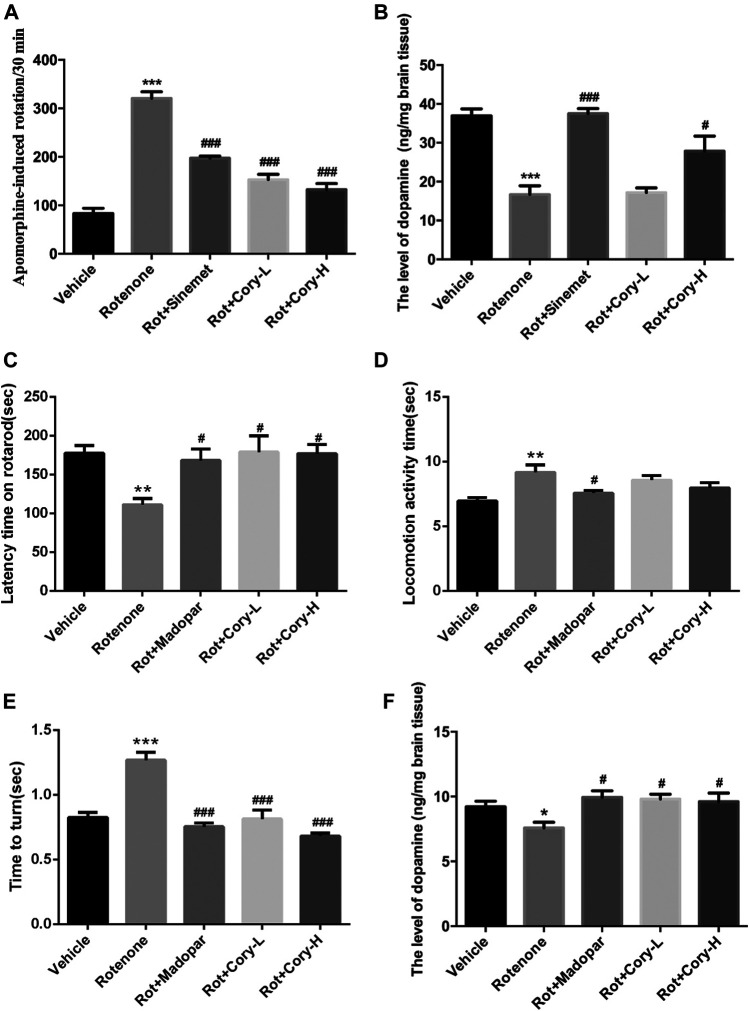
Cory improves the motor dysfunction and increases the striatal dopamine level in the rotenone-induced rat and mice models. **(A–B)** Tests in the rotenone-induced rat model of PD, and **(C–F)** tests in the rotenone-induced mice model of PD. **(A)** Rotation test induced by apomorphine (2.5 mg/kg) was performed at the end of the fourth week after rotenone rat model establishment. Rotations of 30 min were totaled. Cory increases the level of dopamine in the striatum in both of the PD rat **(B)** and mice **(F)** models. In the rotarod test, Cory increases the movement time of rotenone-induced PD mice maintained on the rotarod **(C)**. In the pole test, although the effect of Cory to shorten the locomotion activity time is not significant **(D)**, Cory significantly shortens the turning time **(E)**. Dosage of Cory in the rotenone-induced rat model of PD: Cory-L: 2.5 mg/kg, Cory-H: 5 mg/kg. Dosage of Cory in the rotenone-induced mice model of PD: Cory-L: 5 mg/kg, Cory-H: 10 mg/kg. Data were presented as mean ± SEM. (*p < 0.05, ***p < 0.001 compared with the Sham or Vehicle group, ^#^ *p* < 0.05, ^###^ *p* < 0.001 compared with the Rotenone group, *n* ≥ 6; one-way ANOVA with Newman–Keuls’ multiple test.)

Apomorphine is a nonselective dopamine agonist that activates both D1-like and D2-like receptors, and apomorphine-induced rotation in a rat model of PD is usually used to estimate the motor impairment (Xiong et al., 2009; Carbone et al., 2019; Gunaydin et al., 2019). At the end of the fourth week after rotenone-induced rat models of PD were established, a rotation test induced by apomorphine was performed. Totally, 2.5 mg/kg of apomorphine (i.p.) was intraperitoneally injected, and 35 min of video footage was recorded for each rat. After 5 min of adaptation, rotations were calculated for 30 min. In the rotenone-induced rat model group, the number of rotations induced by apomorphine significantly increased and reached 320 per 30 min. Similar to Sinemet® treatment, both low and high doses of Cory significantly decreased the rotations (Figure 1A). At the end of the eighth week after rotenone-induced mouse models of PD were established (chronic toxicity models), both the rotarod test and the pole test were performed, and the latency time on the rotarod, locomotion activity time, and time to turn on the pole were recorded. The movement time on the rotating rod was significantly shortened in the rotenone group than in the vehicle control group (Figure 1C), and both the climbing time and turning time on the pole were significantly longer in the rotenone group than in the vehicle control group (Figure 1D). Confirming its validity as a widely used drug for the treatment of PD, treatment in clinic, Madopar® significantly improved the motor dysfunction induced by rotenone as shown by the increased latency time on the rotarod, decreased locomotion activity time, and decreased turning time (Figures 1C,D). Cory also contributed toward motor improvements with increased latency time on the rotarod and decreased turning time, although there was no significant difference in the locomotion activity time between the Cory-treated and rotenone groups (Figures 1C–E). At the same time, striatal dopamine levels decrease in both rotenone-induced rat and mouse models (Figures 1B–F). Supplementary of levodopa, which is a precursor of dopamine, significantly increased the dopamine levels in the striatum of the animals in the Sinemet® and Madopar® groups compared to the levels in the animals in the rotenone groups. In addition, a high dose of Cory increased the striatal dopamine levels in the rotenone-induced rat models of PD (Figure 1B), while both the low and high doses of Cory increased striatal dopamine levels in the rotenone-induced mouse models of PD (Figure 1F).

### Effects of Cory on Tyrosine Hydroxylase-Positive Neuronal Loss in the Rotenone-Induced Animal Models of PD

**FIGURE 2:**
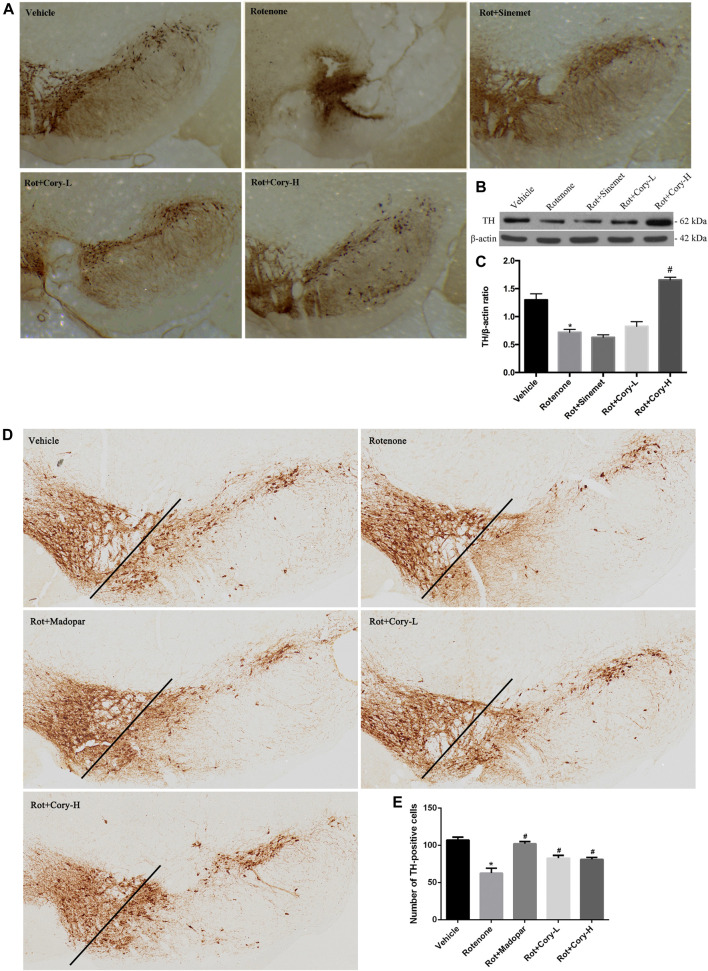
Cory prevents the loss of tyrosine hydroxylase–positive neurons in the SNpc. **(A–C)** Tests in the rotenone-induced rat model of PD, and **(D–E)** tests in the rotenone-induced mice model of PD. Immunostaining results reveal that Cory prevents the loss of tyrosine hydroxylase–positive neurons in the SNpc of the PD rat **(A)** and mice **(D–E)** models. **(B, C)** Cory increases the level of tyrosine hydroxylase. The right side of SNpc was isolated from the brain tissue of the PD rat model. After protein extraction, samples were subjected to Western blotting assay. Dosage of Cory in the rotenone-induced rat model of PD: Cory-L: 2.5 mg/kg, Cory-H: 5 mg/kg. Dosage of Cory in the rotenone-induced mice model of PD: Cory-L: 5 mg/kg, Cory-H: 10 mg/kg. Data were presented as mean ± SEM. (*p < 0.05 compared with Sham or Vehicle group, ^#^ *p* < 0.05 compared with the Rotenone group, *n* ≥ 6; one-way ANOVA with Newman–Keuls’ multiple test.)

TH is the enzyme responsible for catalyzing the conversion of the amino acid L-tyrosine to L-3, 4-dihydroxyphenylalanine (Nagatsu, 1995). It is a rate-limiting enzyme that controls the first step of dopamine biosynthesis. The expression of TH in the right-side brain tissue of SNpc was detected by Western blotting, and the quantification density of TH was analyzed by image J. Lower expression of TH was observed in the right side of SNpc in the rotenone group than in the vehicle group (Figures 2B,C), while Cory significantly increased the levels of TH in these models (Figures 2B,C). Consistent with the Western blotting results, significant loss of TH-positive neurons was found in the right-side brain tissue of the SNpc and striatum in the rotenone-induced rat models (Figure 2A, Supplementary Figure S1). However, the loss of TH-positive neurons in the SNpc and striatum was diminished by treatment with Cory (Figure 2A, Supplementary Figure S1). In the rotenone-induced chronic toxicity mouse models of PD, immunostaining results revealed that rotenone also induced a TH-positive neuronal loss in the SNpc compared to the vehicle group, and Madopar® and Cory showed neuroprotective effects that prevented TH-positive neuronal loss (Figures 2D–E).

### Effect of Cory on Autophagy Induction and Neuroinflammation in Rotenone-Induced Animal Models of PD

**FIGURE 3:**
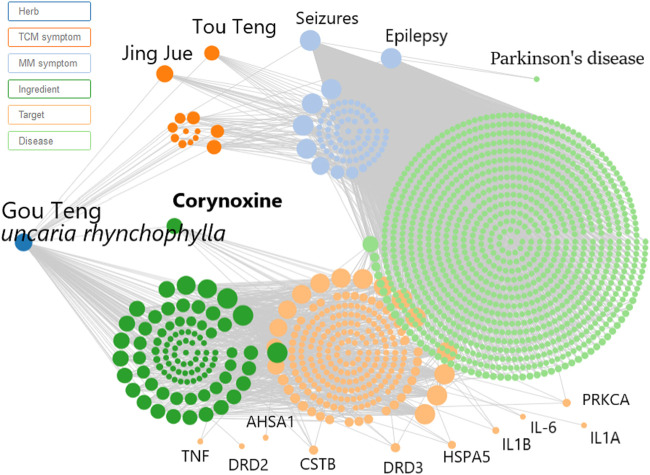
Network pharmacology predicts the mechanism of Cory in treating Parkinson’s disease. Inputting corynoxine into the SymMap (http://www.symmap.org/) database, we got 130 symptoms and 1700 targets. The presented symptoms and targets were screened by this network. And we selected the symptoms of Parkinson’s disease and targets involved in autophagy and neuroinflammation.

Prior to conducting the neuroprotective mechanism study, an herbal search of Cory was performed on the SymMap database (http://www.symmap.org/). The search generated 130 Cory-related symptoms and 1700 targets. Since it has been deduced that Cory can promote the clearance of α-synuclein through the autophagy pathway in a cell model of PD, the symptoms of PD and targets involved in autophagy, including CSTB, HSPA5, PRKCA, and AHSA1, as well as targets involved in neuroinflammation, including TNF, IL-6, IL1B, and IL1A, were all selected from the SymMap integrated network, which indicated the neuroprotective mechanism of Cory in PD (Figure 3).

**FIGURE 4:**
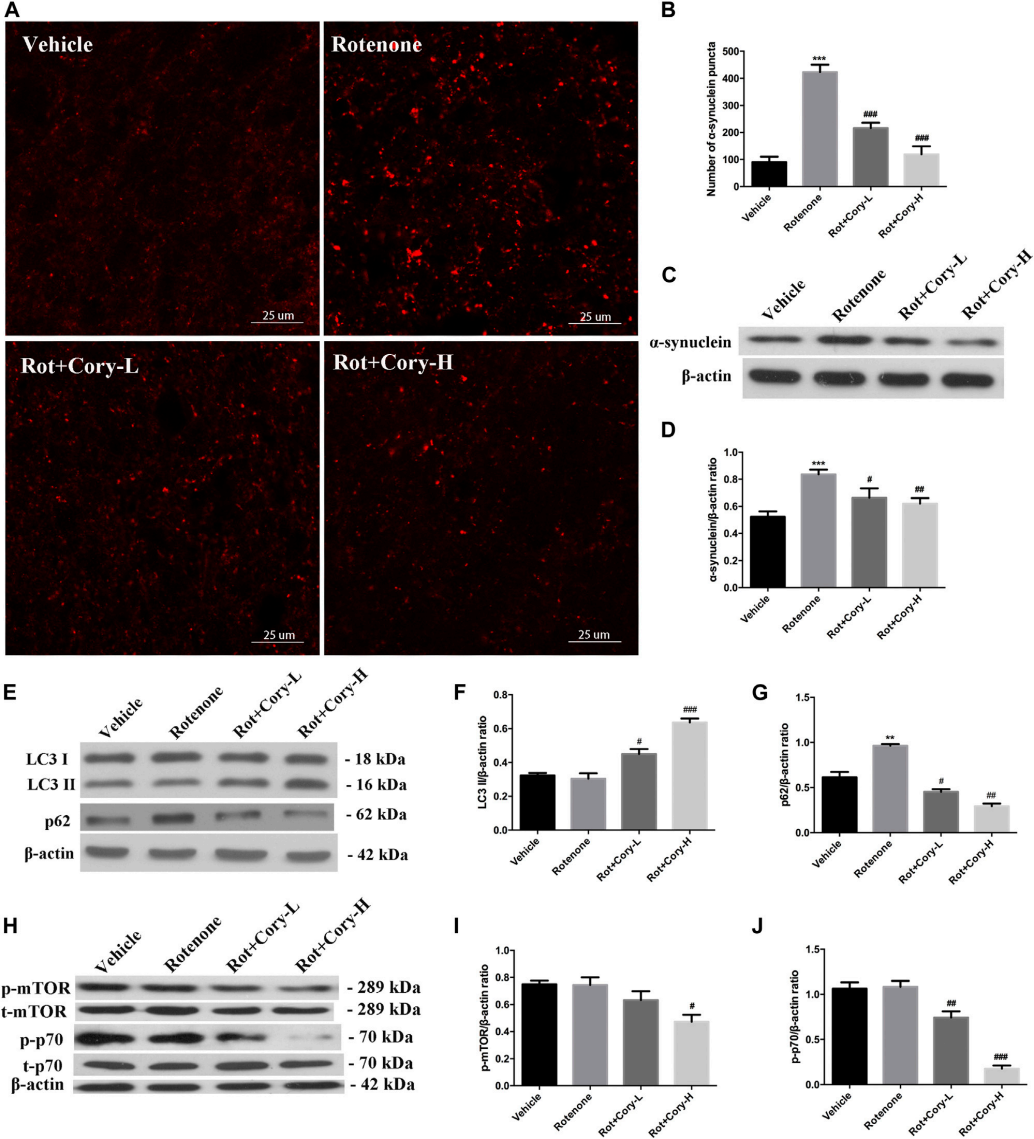
Cory induces autophagy *via* the mTOR pathway and promotes the clearance of α-synuclein aggregation in the rotenone-induced rat model of PD. **(A, B)** Cory decreases the number of α-synuclein aggregations in the right side of SNpc. **(C, D)** Cory decreases α-synuclein expression in the right side of SNpc. The right side of SNpc was isolated from the brain tissue. After protein extraction, samples were subjected to Western blotting assay. **(E–G)** Cory increases LC3 II level and decreases p62 level in the right side of SNpc. **(H–J)** Cory decreases p-mTOR and p-p70 levels in the right side of SNpc. The right side of SNpc was isolated from the brain tissue. After protein extraction, samples were subjected to Western blotting assay. Dosage of Cory in the rotenone-induced rat model of PD: Cory-L: 2.5 mg/kg, Cory-H: 5 mg/kg. Data were presented as mean ± SEM of three independent experiments. (***p < 0.001 compared with Sham model, ^#^ *p* < 0.05, ^#^ *p* < 0.01, ^###^ *p* < 0.001 compared with Rotenone group, one-way ANOVA with Newman-Keuls multiple test.)

In the acute toxicity rat models of PD induced by rotenone, immunostaining results on the brain tissue revealed that the number of α-synuclein aggregates significantly increased in the right side of the SNpc, while the number of α-synuclein aggregates significantly decreased after treatment with Cory (Figures 4A,B). The expression levels of α-synuclein in the right-side brain tissue of SNpc, which were detected by Western blotting, were consistent with the immunostaining results (Figures 4C,D). However, no significant aggregates of α-synuclein were found in the striatum of rats (Supplementary Figure S2). Moreover, the autophagy marker proteins, including LC3 and p62, were detected using Western blotting, with significantly increased p62 levels being observed in the right-side tissue of SNpc in the rotenone group (Figures 4E,G). When compared to the vehicle or rotenone group, Cory increased the level of LC3II and decreased the levels of p62, indicating that Cory was responsible for the induction of the autophagy pathway (Figures 4E–G, Supplementary Figure S3). Furthermore, decreased p-mTOR and p-p70 levels (Figures 4H–J) were observed in the Cory treatment groups, indicating that Cory induces autophagy through the mTOR pathway in the rotenone-induced rat models of PD.

**FIGURE 5:**
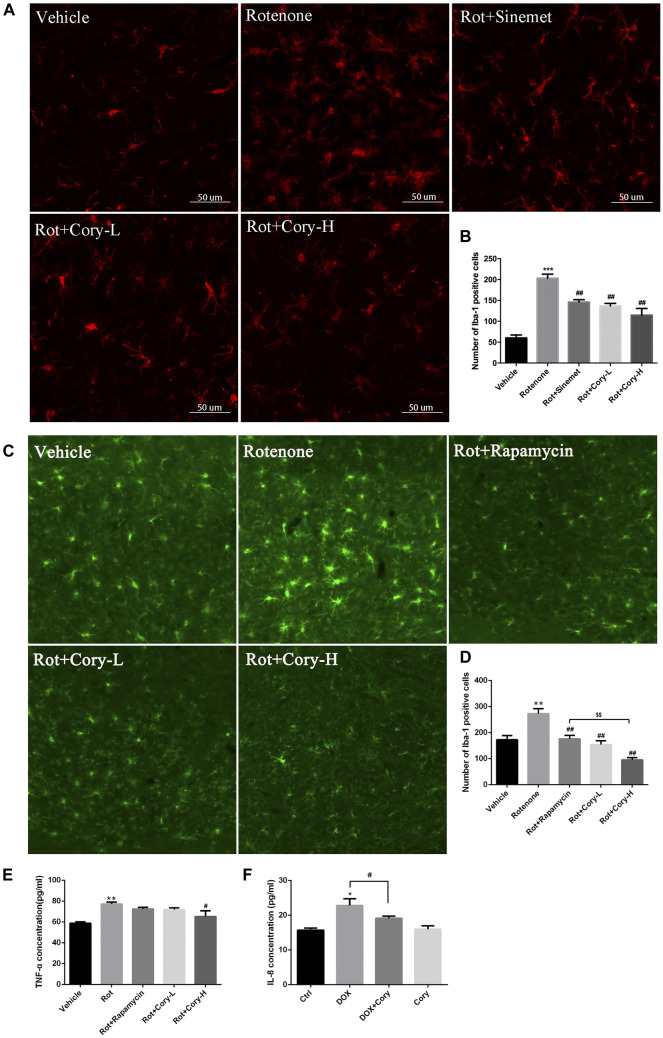
Cory decreases the neuroinflammation *via* inhibiting the activation of microglia and decreasing TNF-α. Cory significantly decreases the number of active microglia cells both in the right-side SNpc of the PD rat model **(A, B)** and in the SNpc of the PD mice model **(C, D)**. **(E)** In the rotenone-induced mice model of PD, Cory significantly decreases the level of TNF-α in the serum. **(F)** Cory diminishes the release of IL-8 in inducible PC12 with overexpression of mutant α-synuclein (A53T). Inducible PC 12 cells were treated with doxycycline for 24 h and further treated with Cory (25 μM) for another 24 h. Supernatant was collected, and the level of IL-8 was analyzed by ELISA. Dosage of Cory in the rotenone-induced rat model of PD: Cory-L: 2.5 mg/kg, Cory-H: 5 mg/kg. Dosage of Cory in the rotenone-induced mice model of PD: Cory-L: 5 mg/kg, Cory-H: 10 mg/kg. Data were presented as mean ± SEM. (**p < 0.01, ***p < 0.001 compared with Sham or Vehicle group; ^#^ *p* < 0.05, ^##^ *p* < 0.01 compared with Rotenone group; ^$$^ *p* < 0.01, Rot + Rapamycin *v*.*s*. Rot + Cory-H; *p <0.05 compared with Ctrl, ^#^ *p* <0.05 compared with Dox; *n* ≥ 6, one-way ANOVA with Newman–Keuls’ multiple test.)

Neuroinflammation, which is characterized by the activation of glial cells and release of pro-inflammatory cytokines, is considered as a detrimental factor in PD. In this study, immunostaining was performed on the brain sections from the SNpc region using the Iba-1 antibody, a marker of microglial cells. The rotenone groups of the two PD models had significantly higher number of active microglial cells than the Vehicle groups (Figures 5A–D). However, after treatment with Cory, the active microglial cells were in the former groups (Figures 5A–D). The level of pro-inflammatory cytokines that were indicated in Figure 3, such as TNF and IL-6, was also detected. In the rotenone-induced chronic toxicity mouse models, the levels of TNF-α significantly increased in the rotenone group, and a high dose of Cory decreased the levels of TNF-α in the serum (Figure 5E). Furthermore, overexpression of α-synuclein induced by doxycycline increased the release of IL-8, and Cory was shown to inhibit an inflammatory response induced by doxycycline in inducible PC12 cells (Figure 5F). However, no significant change of IL-6 release was observed in the inducible PC12 cells (Supplementary Figure S4). The aforementioned results indicate that Cory diminishes neuroinflammation in the mouse and cell models of PD.

## Discussion

In the year 2000, rotenone was first reported to show features of PD following chronic systemic exposure (Betarbet et al., 2000). These effects were reported to be similar to those produced by 1-methyl-4-phenyl-1,2,3,6-tetrahydropyridine (MPTP), and since then, animal models induced by rotenone have been investigated. Due to the lipophilic nature, rotenone crosses the blood–brain barrier and cell membranes with relative ease in comparison to MPTP. The behavioral, central, and peripheral neurodegenerative features of PD, such as dopaminergic neuronal cell loss in the SNpc, lesioned nerve terminals in the striatum, as well as increased α-synuclein aggregates in the SNpc, dorsal motor nucleus of the vagus, and intestinal myenteric plexus, are all well reproduced by rotenone treatment (Miyazaki et al., 2020). However, the PD model established with systemic administration of rotenone usually caused a high mortality rate and various neuropathological changes (Richardson et al., 2019). To avoid these disadvantages of mouse models with systemic administration of rotenone, a rat model with stereotaxical infusion of rotenone into the SNpc was also employed in this study. Two safe doses of Cory were also administered to the animals to evaluate the neuroprotective properties of the alkaloid.

Due to the dopaminergic neuronal impairment induced by rotenone in the SNpc and striatum, motor dysfunction symptoms, evaluated by apomorphine-induced rotation, the rotarod test, and the pole test, were present in the rotenone-treated rats and mice (Figures 1, 2). Similar to Sinemet® and Madopar® treatment, Cory increased dopamine levels in the striatum and latency time on the rotarod, and decreased the rotations induced by apomorphine and turning time in the pole test, indicating that Cory may play a protective role in motor dysfunction by increasing dopamine (Figure 1). Dopaminergic neuronal loss and significantly decreased TH levels in the SNpc were reflected in the rotenone-treated rats or mice (Figure 2). However, Cory treatments had a neuroprotective effect by preventing the TH-positive neuronal loss and increasing TH levels (Figure 2). All these data indicate that Cory protects the nigra-striatum dopaminergic system and improves the motor dysfunction and dopaminergic neuronal loss induced by rotenone.

The genus *Uncaria* is an important folk medicine to be wildly used in China, Malaysia, the Philippines, Africa, and Southeast America (Zhang et al., 2015). Up to now, more than 200 compounds, including indole alkaloids, triterpenes, flavonoids, phenols, and phenylpropanoids, have been isolated from *Uncaria*. As the characteristic constituents, indole alkaloids isolated from *Uncaria* are reported to be efficacy for PD, Alzheimer’s disease, hypertension, and epilepsy, and depressant (Zhang et al., 2015; Zhao et al., 2019a; Zhao et al., 2019b; Zheng et al., 2020; Zhao et al., 2021). Cory is an oxindole alkaloid isolated from *Uncaria rhynchophylla*. Previously, we provided evidences that Cory induced autophagy and promoted the clearance of α-synuclein through the Akt/mTOR pathway in neuronal cells, including N2a, SH-SY5Y, and PC12 cells. Meanwhile, Cory induced autophagy in Cg-GAL4 > UAS-GFP-Atg8a *Drosophila* larvae fat body (Chen et al., 2014). In order to identify the key protein kinase, a novel network-based algorithm of *in silico* kinome activity profiling, which was named iKAP, was developed, and MAP2K2 was found to play an essential role in the progress of Cory-induced autophagy (Chen et al., 2017; Chen et al., 2018; Chen and Xie, 2018b). In this study, increased α-synuclein aggregates were found in the lesioned side of the SNpc in the rotenone group (Figures 4A–D), which is consistent with previous reports (Ramalingam et al., 2019; Richardson et al., 2019). Decreased p62, a substrate of autophagy, and increased LC3II indicated that the aforementioned alkaloid induced autophagy in the rotenone rat models of PD (Figures 4E,G). Furthermore, decreased levels of p-mTOR and p-p70 indicated that Cory induced autophagy through the mTOR pathway (Figures 4H–J) in the animal models, which is consistent with the results obtained from cell modes. Induction of autophagy by Cory, which is considered to be an effective route to clear aggregated α-synuclein, resulted in a significant decrease in the aggregation and expression of α-synuclein after Cory treatment (Figures 4A–D). Therefore, Cory may promote the clearance of α-synuclein *via* autophagy in a rotenone-induced rat model of PD.

Recently, neuroinflammation, which is induced by the activation of microglia and the release of pro-inflammatory cytokines, was reported to play an important role in the degeneration of dopaminergic neurons in PD (Ho, 2019; Liu et al., 2019). In patients who were at early stages of PD, both microglial activation and dopaminergic terminal loss were observed in the midbrains and thalamus (Ouchi et al., 2005). Heightened levels of pro-inflammatory cytokines, such as TGF-beta1, IL-6, and IL-1β, were also found in the cerebrospinal fluid of these PD patients (Chen et al., 2018). A rat model of PD with overexpression of human α-synuclein displayed microglial activation and neuroinflammation, which were coupled with early alterations, including reduced striatal dopamine outflow and motor dysfunction, prior to nigral degeneration. Prevention of the central and peripheral inflammation by resolving D1 improves the neuronal dysfunction and motor deficits (Krashia et al., 2019). Recent reports have shown that microglia can clear the α-synuclein released by neurons through TLR4-NF-kappaB-p62–mediated synucleinphagy (Choi et al., 2020). In the present study, both the activated microglia and increased pro-inflammatory cytokines induced by rotenone, as well as increased IL-8 levels induced by α-synuclein overexpression, were diminished by Cory (Figure 5). In addition, the effects of Cory on microglia activation and TNF-α secretion were better than those of rapamycin, which has been reported to suppress microglial activation and TNF-α expression induced by intracerebral hemorrhage (Li et al., 2016; Karunakaran et al., 2019). All these reports support the link between microglia and α-synuclein in contributing to dopaminergic degeneration in PD. However, the potential anti-neuroinflammatory effects of Cory highlighted in this study may also be responsible for its neuroprotective abilities in PD.

Collectively, these study findings indicated that Cory possessed neuroprotective effects in rotenone-induced rat and mouse models of PD by improving motor dysfunction, preventing TH-positive neuronal loss, decreasing α-synuclein aggregates through the mTOR pathway, and diminishing neuroinflammation. This provides experimental data to support the development of Cory for the treatment of PD.
