# Supplementary figures and images for: Corynoxine Protects Dopaminergic Neurons Through Inducing Autophagy and Diminishing Neuroinflammation in Rotenone-Induced Animal Models of Parkinson’s Disease
Source: Front Pharmacol. 2021 Apr 13;12:642900. doi: 10.3389/fphar.2021.642900 (PMC8078868; doi:10.3389/fphar.2021.642900)

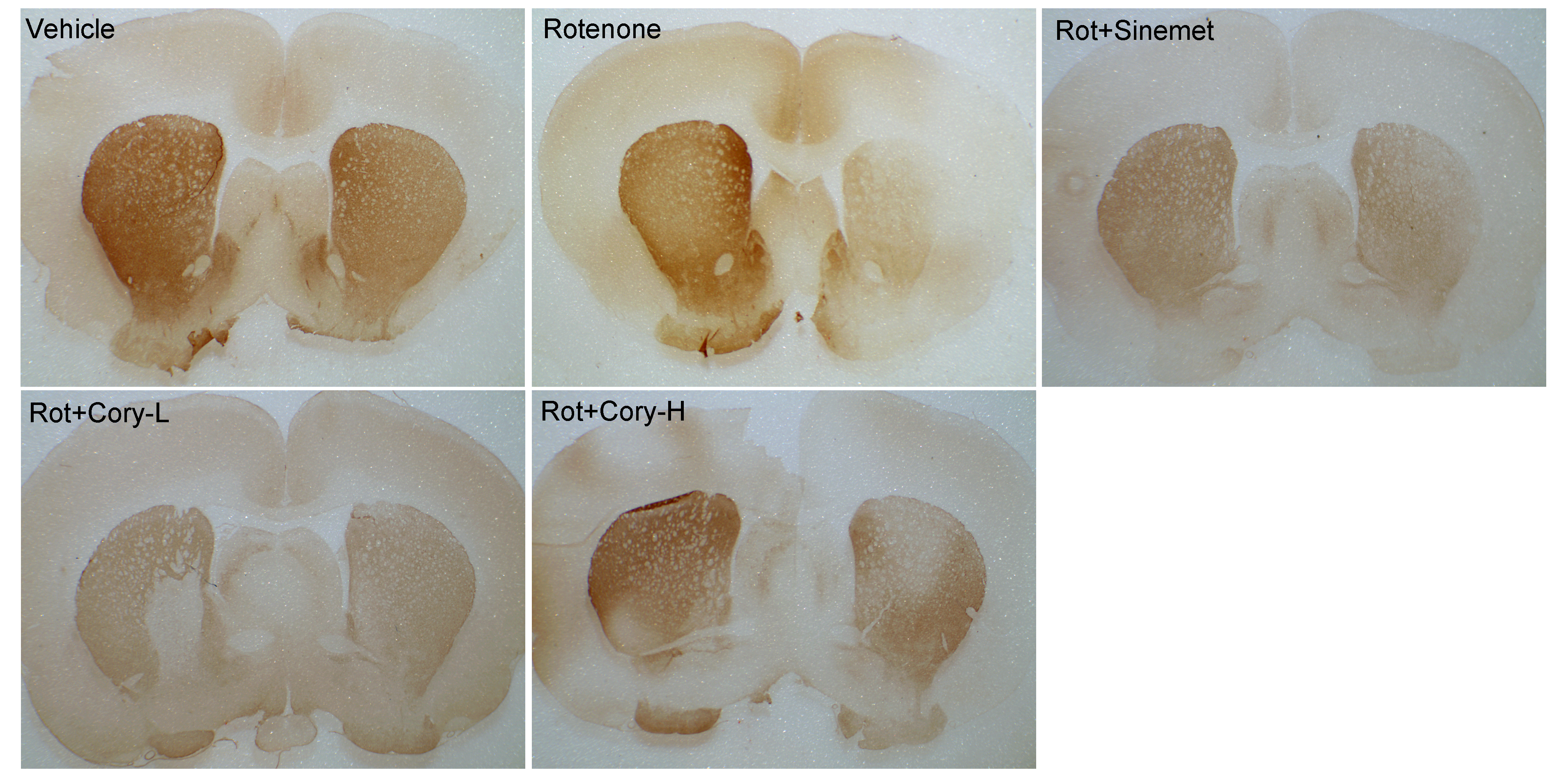

Supplement: Supplementary file 1 [file image1.jpeg]

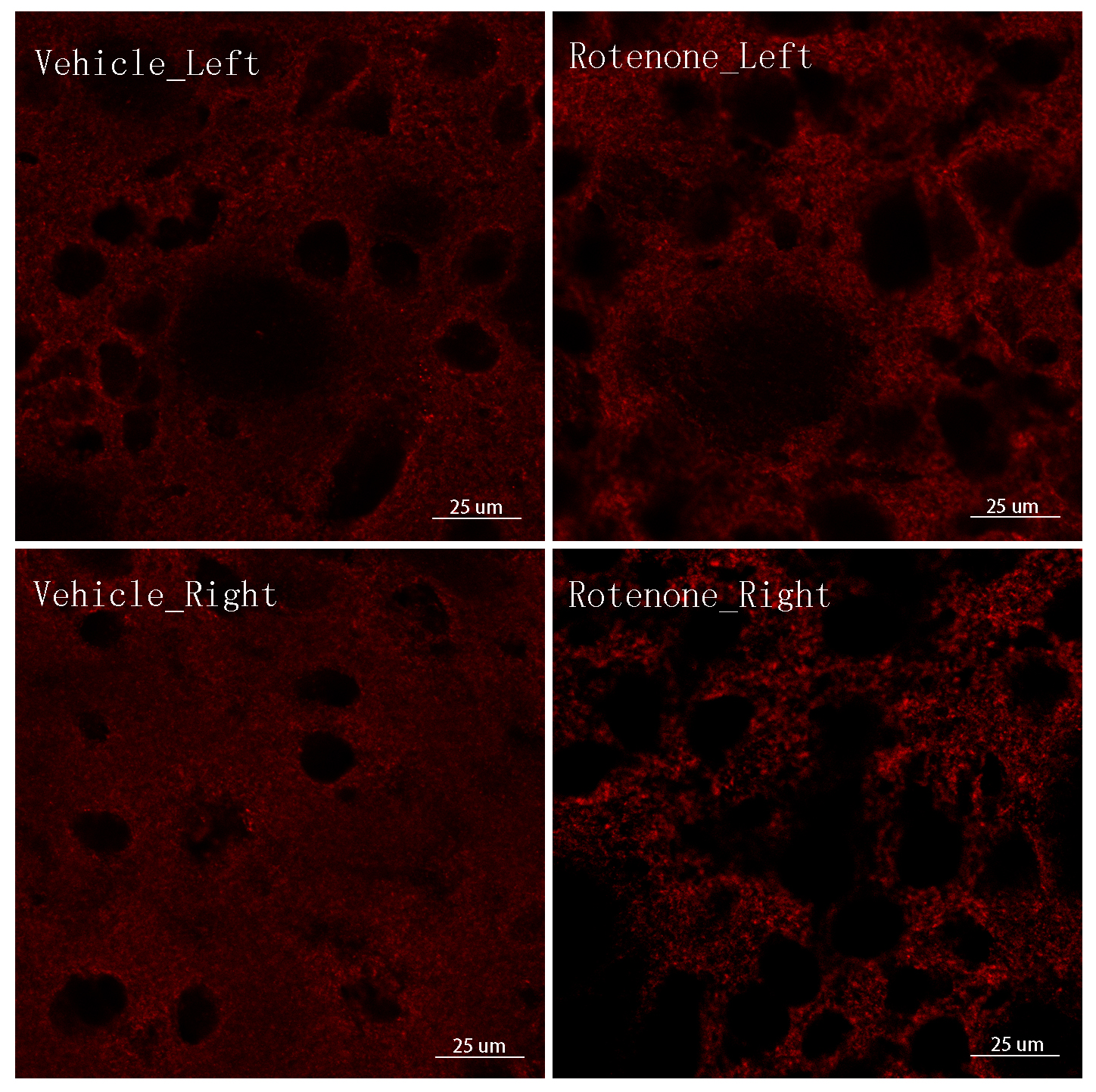

Supplement: Supplementary file 2 [file image2.jpeg]

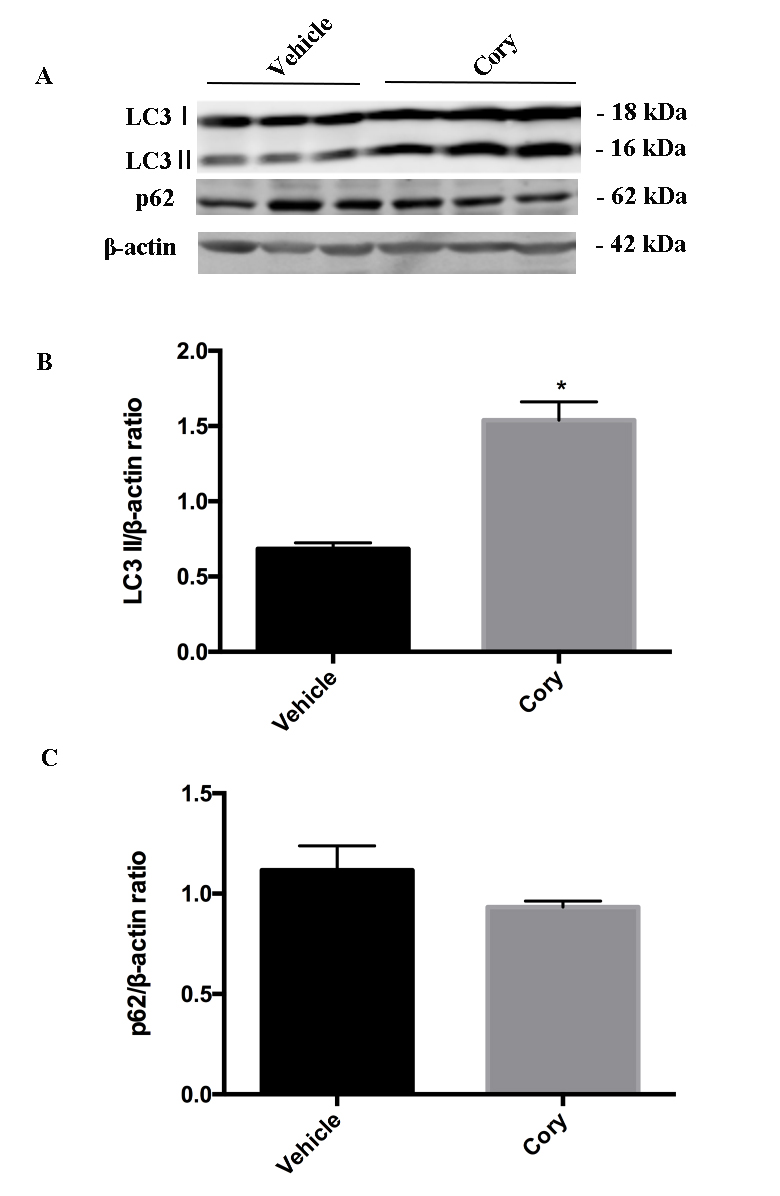

Supplement: Supplementary file 3 [file image3.jpeg]

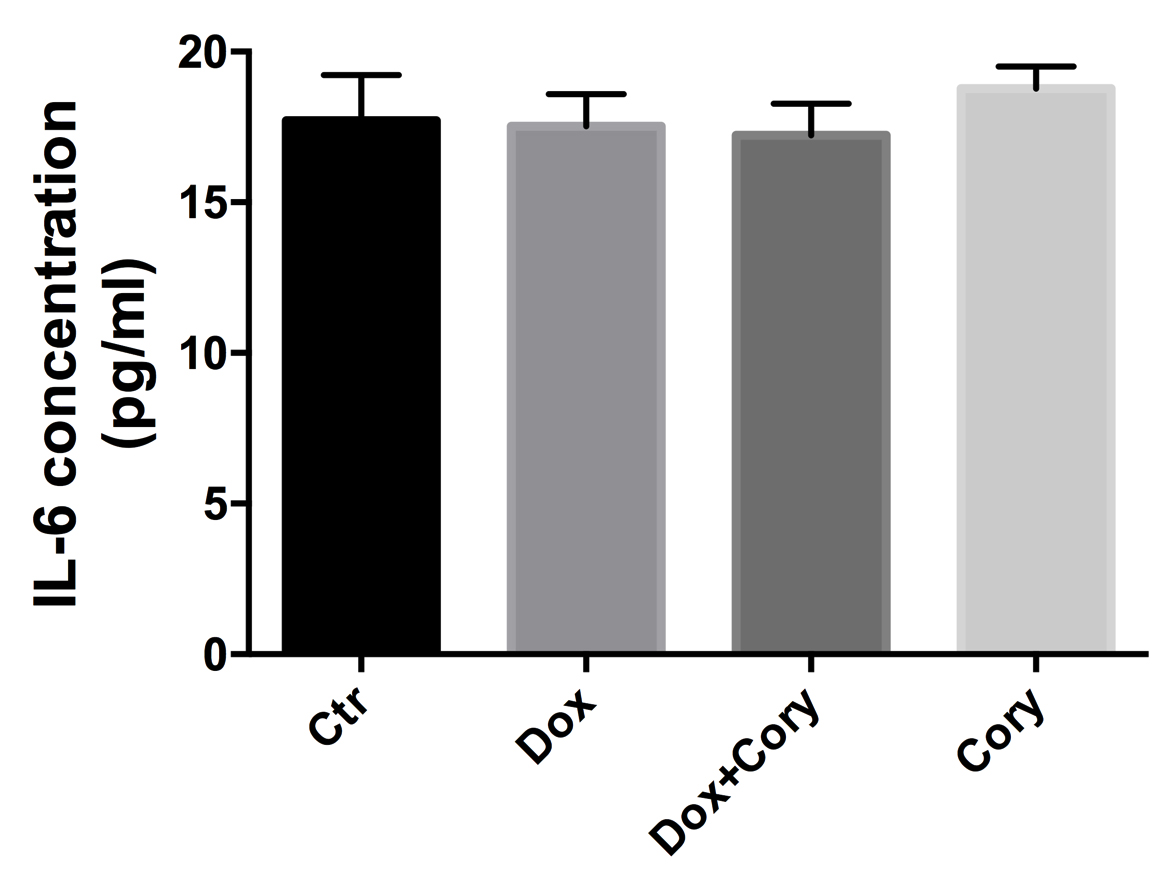

Supplement: Supplementary file 4 [file image4.jpeg]
